# Performance Enhancement in Federated Learning by Reducing Class Imbalance of Non-IID Data

**DOI:** 10.3390/s23031152

**Published:** 2023-01-19

**Authors:** Mihye Seol, Taejoon Kim

**Affiliations:** School of Information and Communication Engineering, Chungbuk National University, Chungju 28644, Republic of Korea

**Keywords:** federated learning, non-IID data, class imbalance mitigation

## Abstract

Due to the distributed data collection and learning in federated learnings, many clients conduct local training with non-independent and identically distributed (non-IID) datasets. Accordingly, the training from these datasets results in severe performance degradation. We propose an efficient algorithm for enhancing the performance of federated learning by overcoming the negative effects of non-IID datasets. First, the intra-client class imbalance is reduced by rendering the class distribution of clients close to Uniform distribution. Second, the clients to participate in federated learning are selected to make their integrated class distribution close to Uniform distribution for the purpose of mitigating the inter-client class imbalance, which represents the class distribution difference among clients. In addition, the amount of local training data for the selected clients is finely adjusted. Finally, in order to increase the efficiency of federated learning, the batch size and the learning rate of local training for the selected clients are dynamically controlled reflecting the effective size of the local dataset for each client. In the performance evaluation on CIFAR-10 and MNIST datasets, the proposed algorithm achieves 20% higher accuracy than existing federated learning algorithms. Moreover, in achieving this huge accuracy improvement, the proposed algorithm uses less computation and communication resources compared to existing algorithms in terms of the amount of data used and the number of clients joined in the training.

## 1. Introduction

As the number of smartphones and Internet of Things (IoT) devices grows rapidly, the amount of data they are generating is growing explosively [1]. A mainstream in utilizing this large volume of data distributed over multiple devices is centralized data processing, i.e., transferring those devices’ data to a server and training a machine learning model from it. However, transferring this huge amount of data to the processing server causes network overhead and increases communication costs. Additionally, data processing servers demand enormous storage and computing power, resulting in high maintenance costs. Federated learning (FL) has been proposed to solve these problems [2].

FL allows clients to cooperate to generate a global model without sharing the clients’ data with a server. Federated Averaging (FedAVG) [3], a representative algorithm of FL, sends the local model parameters to a server after each device learns a local model using its own local dataset. The server configures a global model by aggregating the received local parameters. However, unlike central data processing, FL uses clients’ resources to learn models, accordingly, the system heterogeneity (computing power, wireless channel environment, size of dataset, etc.) among clients has a significant impact on learning efficiency.

To resolve the problem of system heterogeneity among clients, a lot of research works were conducted to schedule devices on servers. In [4], the authors proposed a method of selecting clients based on the available amount of communication and computing resources with the goal of fast convergence and high accuracy of a global model. The methods of effectively utilizing communication resources were proposed in [5,6]. In [5], a method of controlling the frequency between a global aggregation and a local update was proposed. In [6], the authors proposed a method of applying hierarchical aggregation. In [7], the model parameters for servers and local clients were compressed for efficient use of communication resources. In addition, some research works were conducted to dynamically allocate batch size for clients based on the available amount of communication and computing resources [8,9,10].

One of the most important issues in FL is statistical heterogeneity, i.e., the negative effect of non-independent and identically distribution (non-IID) of the training dataset. The distribution of data generated by a client varies depending on the client’s occupation, lifestyle, residential area, etc. As a result, the local data distribution of a client will be non-IID with a high probability. Accordingly, the class distribution of the client also has a class imbalance.

Class imbalance can be categorized into intra-client class imbalance and inter-client class imbalance. Intra-client class imbalance means that the distribution of data amount among classes, i.e., class distribution, in a client is different from Uniform distribution. The larger the distribution gap is, the more severe the imbalance is. Inter-client class imbalance means that the class distribution of each client is different from other clients’ distribution. In [11,12], it was confirmed that the accuracy of FL was decreased when these intra- and inter-client class imbalances were considered.

Although a lot of research works have been conducted to prevent learning efficiency from decreasing when the class distribution of clients is imbalanced, to the best of our knowledge, an integrated research work incorporating three core components—(1) a method of reducing the intra-client class imbalance, (2) a method of reducing the inter-client class imbalance, and (3) a method of dynamic batch size allocation and learning rate control—has never been conducted.

We propose a novel algorithm that supports intra- and inter-client class imbalance mitigation and dynamic batch size allocation and learning rate control considering the amount of local dataset. First, the proposed algorithm performs data oversampling to make the class distribution of each client close to Uniform distribution. This oversampling scheme for FL, to the best of our knowledge, is the first approach incorporating an exponential decay factor, and it dynamically reflects the amount of oversampled data in the previous round. Second, to avoid performance degradation due to inter-client class imbalance, the clients to join FL are selected to balance the aggregated class distribution for each round, and the amount of data to be actually used for local learning is also adjusted by considering the class distributions of the selected clients. The combination of these two features in client selection is a unique contribution of this paper and shows significant performance improvement. Finally, the batch size and the learning rate of the selected clients are adjusted according to the amount of data for the clients. It is also the first approach presenting the dynamic batch size and learning rate adjustment assuming a common SGD update in an FL.

The performance of the proposed algorithm is validated over the CIFAR-10 [13] and MNIST [14] datasets in non-IID scenarios, and it is confirmed that the accuracy of the global model from the proposed algorithm achieves about 20% better performance than existing FL algorithms in non-IID situations. Moreover, despite this remarkable improvement in accuracy, the computing and communication resource usage in terms of the amount of data used for learning and the number of clients participating in learning are decreased compared to existing FL algorithms.

The main contributions of this paper are summarized as follows:

- To mitigate intra-client class imbalance, a novel data sampling to local datasets is introduced, which results in accuracy improvement in non-IID environments.
- An FL server intelligently selects clients and allocates the amount of data to be actually used in local learning by balancing the class distributions of the selected clients.
- The batch size and the learning rate of clients are dynamically controlled according to the amount of local dataset for each client.
- Performance evaluation in various non-IID scenarios confirms that the proposed algorithm achieves high accuracy and low usage of computing and communication resources compared to existing algorithms.

The remainder of this paper describes the following. Section 2 introduces the literature review, and Section 3 describes the overall system structure and defines the class distribution of clients. Section 4 describes the detailed procedure of the proposed algorithm, Section 5 shows the experimental results, and finally, Section 6 concludes the paper.

## 2. Related Works

In the literature, various research works were conducted to improve the performance of a global model under a non-IID dataset. In an intra-client class imbalance situation, in order to solve the learning efficiency reduction problem, there was an attempt to make the local class distribution of clients close to IID by sharing data among clients. In [12], a small IID dataset was created in a server by collecting data from clients to mitigate the negative effects of intra-client class imbalance. However, this approach does not meet the original purpose of FL because the clients’ privacy is not protected by transmitting their data to the server to generate the small IID dataset.

In [15], both the statistical heterogeneity and the system heterogeneity were considered to prevent local models from deviating from a global model. Specifically, a proximal term was added to a loss function. Similarly, in [16], the elastic weight consolidation method was proposed to add a penalty term to a loss function to prevent the models of non-IID clients from drifting apart from each other.

Another research approach to alleviating inter-client class imbalance is to more intelligently select the clients to participate in an FL. The authors of [17] have improved the performance of FL by increasing the selection probability for the clients having a large gradient value. In [18], a scheme of group learning for clients with similar class distributions and merging the trained models into a global model was proposed. In [19], FL models could converge with fewer rounds through a hierarchical clustering of clients based on the similarity of local models of clients. In [20], the data augmentation scheme was proposed as a solution to a global imbalance situation in which the aggregated class data distribution of clients differs from Uniform distribution. In addition, mediator-based client rescheduling is introduced to alleviate local imbalance.

The level of IID for the local dataset was evaluated using weight divergence and multi-arm bandit [21]-based algorithms in [22] and [23], respectively. Moreover, in [23], the negative effects of local imbalance were reduced by increasing the selection probability of clients with high IID levels. The authors in [24] showed that, for performance improvement, the aggregation weights of local models should be finely adjusted considering the quality and quantity of local data, the number of classes, and the entropy of local data.

In FedNova [25], the performance degradation due to the differences in the number of local updates was reported, and this difference was from the heterogeneity in non-IID local datasets and computation resources. To solve this problem, a normalized model aggregation method was proposed. In [26], the performance degradation of stochastic gradient descent (SGD) method over non-IID data was mitigated by introducing a deep reinforcement learning-based client selection and client-specific batch size allocation scheme.

Although various studies have been conducted until now, research considering both intra- and inter-client class imbalance mitigation and dynamic batch size and learning rate adjustment considering the size of the dataset has not been conducted.

## 3. System Model and Data Distributions

### 3.1. System Model

An FL system for a multi-class classification task consists of a server to manage the global model and a set of clients 𝒦 ={1, 2, …,K}. Each client has a local dataset, and client k’s local dataset is denoted by $D_{k}$. In rth round of the FL, client k, which is selected to participate in this learning, starts a local learning using its local dataset $D_{k}$ with the initial global model vector $w_{r}$, received from the server. Client k makes up a mini batch set ${ℬ}_{k}$ from the local dataset $D_{k}$ and proceeds with the local learning using an SGD optimizer. The update rule for the local learning is expressed as follows:

(1) $$w_{k,r+1}\leftarrow \;w_{k,r}-\eta \frac{1}{\left|D_{k}\right|}\underset{x\in {ℬ}_{k}}{\sum }\nabla f_{k}\left(w_{k,r};x\right),\;\forall k\in K,$$

where $\left|D_{k}\right|$ denotes the cardinality of $D_{k}$, $f_{k}\left(w_{k,r};x\right)$ is a loss function for the local model vector $w_{k,r\;}$ and data x, and η is the learning rate. Each selected client trains the local model until a pre-determined local epoch and transmits the learned local vector to the server. The server updates the global model vector by aggregating the received local model vectors. In the aggregation process, a weight for each local model is required, and it is determined to be the amount of data used in each local training divided by the total amount of data for the entire clients participating in the learning. The aggregation with the weights is given by

(2) $$w_{r+1}\;\leftarrow \;\overset{}{\underset{k\in S}{\sum }}\frac{\left|D{’}_{k}\right|}{\left|D\right|}w_{k,\;r+1},$$

where S denotes the set of clients selected by the server to participate in the learning, $D≜\;\cup_{k\in S}D_{k}$. $D\prime_{k}$ denotes data used by client k for local learning and has a relation of $D_{k}^{\prime }\subset D_{k}$. This process is repeated until a specified round is reached. The main parameters of the system model are summarized in Table 1.

### 3.2. Data Distributions

As shown in [27], the class distribution of a client is set using Dirichlet distribution. When a classification task has L classes to classify, it is assumed that all clients’ local learning data are extracted according to a vector $q\;\left(q_{𝓁}\;\geq 0,\;𝓁\;\in \left[1,L\right]\;\mathrm{and}\;∥q{∥}_{1}=1\right)$, which corresponds to the class distribution. The class distribution of the clients is determined by q ~ Dir(αp) of Dirichlet distribution, where p denotes the prior probability distribution and α is a parameter that adjusts the uniformity of the class distribution among the clients. If α>0 and α→∞, then the class distribution of all the clients approaches to Uniform distribution. Conversely, when α is close to 0, all the clients have only a single class of data, resulting in a non-IID class distribution.

## 4. Proposed Algorithm

### 4.1. Alleviating Intra-Client Class Imbalance

When the clients’ class distribution is IID, the performance of the FL is very close to centralized learning methods. However, when the class distribution of the local dataset is non-IID, the accuracy of the FL decreases because the local model learned is biased to some class data. Hence, the performance of the FL model can be improved when the class distribution of each client is close to Uniform distribution. Consequently, an oversampling method of making the class distribution close to Uniform distribution without data sharing is proposed. The purpose of this scheme is to render the non-IID dataset to IID as close as possible.

Denote $n_{k}={\left[n_{k}^{1},\;n_{k}^{2},\ldots ,n_{k}^{L}\right]}^{T}$ as class data distribution vector for client k, where $n_{k}^{j}$ is the data amount of j-th class for client k. Then, the average amount of data over the classes is $t_{k}=\frac{1}{L}\overset{L}{\underset{𝓁}{\sum }}n_{k}^{𝓁}$. Client k randomly oversamples data elements in the classes having a smaller volume than $t_{k}$. This oversampling is conducted until the volume of each class reaches the average $t_{k}$ ($n_{k}^{𝓁}\;\geq \;t_{k},𝓁\in \left[1,L\right]$). One of the noteworthy features of this oversampling is that it reduces intra-client class imbalance without losing any of the data obtained with much effort. Due to this data reserving characteristics of the oversampling, it outperforms the other method of sampling at the level of an average over classes.

**Figure 1:**
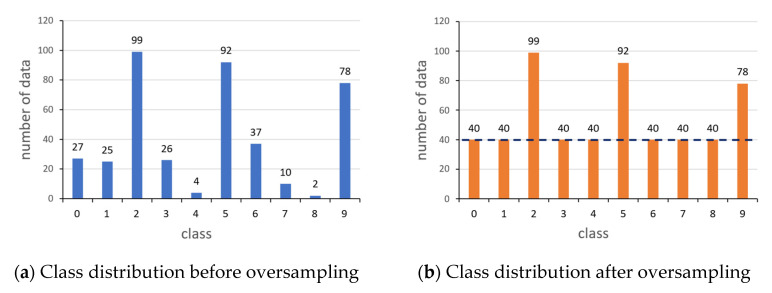
Class data distribution through data oversampling, where the dotted line in (**b**) is the average amount of data per class.

However, note that data oversampling can lead to overfitting, which reduces the generalizability of the model as the amount of local data increases. To avoid overfitting, the amount of oversampled data per round should be reduced as the round goes on. Accordingly, the training load on each client due to the oversampling is diminished rapidly. Specifically, in the proposed method, the amount of oversampling is exponentially reduced as $e^{-\delta r}$, where r is the round index and the exponent δ is increased when the amount of total oversampled data in the previous round is greater than the threshold $\theta_{\mathrm{over}}$. This scheme enables the early termination of the oversampling to prevent the local model from falling into overfitting. Figure 1 shows the local data sampling of a client, and the detailed process is represented in Algorithm 1.
**Algorithm 1.** Sampling. number of data per class is greater than or equal to the average• **client executes:** • **Input** $n_{K}$, r round index, δ oversampling exponent • **Output** $n_{K}$ 1: $t_{k}\leftarrow \frac{1}{L}\overset{L}{\underset{𝓁}{\sum }}n_{k}^{𝓁}\times e^{-\delta r}\;$ 2: **repeat**
3: oversampling for $D_{k}$
4: **until** $n_{k}^{𝓁}\;\geq$ $t_{k},\;𝓁\in \left[1,L\right]$
5: **return**
$n_{K}$ • **server executes:**
• **Input**   S selected client set
• **Output** δ 1: **if** $\frac{\sum_{k\in S}^{k}\left(\sum_{𝓁}^{L}n_{k}^{𝓁}-\left|D_{k}\right|\right)}{\sum_{k\in S}^{k}\left|D_{k}\right|}>\theta_{\mathrm{over}}$ //calculate oversample data rate 2: δ←δ+Δ
3: **return**
δ


### 4.2. Alleviating Inter-Client Class Imbalance

To alleviate the class imbalance among the clients, the server utilizes the sum of the class distributions in selecting the clients to participate in the FL. In addition to this client selection, the server determines the amount of data per class to learn for the selected clients. In each round, the negative effect of inter-client class imbalance can be alleviated by rendering the aggregated distribution of the total training data close to Uniform distribution.

Specifically, any client who wants to participate in rth round learning conducts data oversampling for intra-client class imbalance mitigation and transmits class data volume information $n_{k}$ to the server. The client can transmit the information about the amount of data to contribute to the learning in this round by reflecting it in $n_{k}$. This sharing of $n_{k}$ may reveal the information of the clients to the server; however, note that not the content of data but only the class distribution information is transmitted. Moreover, $n_{k}$ can be different from the actual class distribution of client k. The server manages the data amount information $s_{k,r}={\left[s_{k,r}^{1},s_{k,r}^{2},\;\ldots ,\;s_{k,r}^{L}\right]}^{T}$ for each client k, where $s_{k,r}^{𝓁}$, 𝓁=1, …, L is the amount of 𝓁th class data for client k to learn in rth round. Considering all the selected clients, the server also manages the information of the amount of data per class required in the learning as $v_{r}={\left[v_{r}^{1},v_{r}^{2},\;\ldots ,\;v_{r}^{L}\right]}^{T}$, where $v_{r}^{𝓁},\;𝓁=1,\;\ldots ,\;L$ is the total amount of 𝓁th class data to learn in rth round.

The server sorts the clients willing to participate in the learning in descending order of the amount of local dataset, i.e., $\overset{L}{\underset{𝓁}{\sum }}n_{k}^{𝓁}$. Let client k be on the top of the sorted client list. The server updates $v_{r}$ and $s_{k,r}$ with the data amount information $n_{k}$. In ***v**_r_*, the server finds out the class with a maximum amount of data and the class with a minimum amount of data. Now, assume that the volume of the maximum amount class is denoted as m and the class index for the minimum amount of data is denoted as f. Through the sorted client list, the server searches for a client i having data to learn in class f. Similarly, ***v**_r_* and $s_{i,r}$ are updated using $n_{i}$.

However, in this update process, we regulate the accumulated amount of data in each class to be equal to or smaller than the maximum value m so that this process does not break the balance among the classes. When $v_{r}$ is updated, the uniformity of class distribution $v_{r}$ is tested by calculating the Kullback–Leibler divergence (KLD) [28] between $v_{r}$ and Uniform distribution. This client selection process terminates if the calculated KLD is below the threshold $\theta_{KLD}$, or if the number of selected clients reaches the maximum number of clients h. Finally, the server informs the selected clients of the amount of data to learn by delivering $s_{s,r}$ to client s∈S. This process is expressed in Algorithm 2.
**Algorithm 2.** Client Selection. The server selects clients and adjusts client’s training data• **Input** $K,\;\;\;N=\left\{n_{1},\;n_{2,}\ldots ,\;n_{K}\right\}{\;\mathrm{client}}^{’}s\;\mathrm{class}\;\mathrm{information}\;\mathrm{set},\;\theta_{KLD}\;\mathrm{KLD}\;\mathrm{threshold},\;h\;\;\mathrm{maximum}\;\mathrm{number}\;\mathrm{of}\;\mathrm{selected}\;\mathrm{client},L\;\mathrm{number}\;\mathrm{of}\;\mathrm{classes}$ • **Output** selected client class information set $S_{\mathrm{info}}$ = ${\left\{s_{1,r},\;s_{2,r,}\ldots ,\;s_{h,r}\right\}}^{T}$ 1: **initialize**
$S_{\mathrm{info}}\leftarrow \emptyset ,\mathrm{data}\;\mathrm{volume}\;\mathrm{vector}\;\;v_{r}={\left[v_{r}^{1},v_{r}^{2},\;\ldots ,\;v_{r}^{L}\right]}^{T}$ 2: Sort N in descending order by the amount of data $\overset{L}{\underset{𝓁}{\sum }}n_{k}^{𝓁},\;k\in N$ 3: **repeat** 4:   **for each**
$n_{c}\in N$
**do** 5:    **if** $S_{\mathrm{info}}\;\mathrm{is}\;\mathrm{empty}$ **then** 6:     **for each** 𝓁, 𝓁 = 1, 2, …, L
**do** 7:      $v_{r}^{𝓁}\;\leftarrow v_{r}^{𝓁}+n_{c}^{𝓁}$ 8:      $s_{c,r}^{𝓁}$ $\leftarrow n_{c}^{𝓁}$
9:       **end for** 10:     m ← max($v_{r}$) //Maximum value among $v_{r}$ 11:     add $s_{c,r}$ in $S_{\mathrm{info}}$ 12:    **else** 13:     f← ${\mathrm{argmin}}_{𝓁}\;\left(v_{r}\right)$ 14:     **if**
$n_{c}^{f}>0$ **then**
15:      **for each**
𝓁, 𝓁 = 1, 2, …, L **do** 16:       $v_{r}^{𝓁}\leftarrow$ $v_{r}^{𝓁}$
**+** min(m—$v_{r}^{𝓁}$, $n_{c}^{𝓁}$) 17:       $s_{c,r}^{𝓁}$ ← min(m—$v_{r}^{𝓁}$, $n_{c}^{𝓁}$) 18:      **end for** 19:      add $s_{c,r}$ in $S_{\mathrm{info}}$ 20:     **end if** 21:    **end if** 22:   **end for**
23: **until**
$\left|S_{\mathrm{info}}\right|==h\;\mathrm{or}\;D_{KL}\;\left(P_{v_{r}}│P_{\mathrm{uniform}}\right)<\theta_{KLD}$ 24: **return**
$S_{\mathrm{info}}$


### 4.3. Dynamic Batch Size and Learning Rate Control

In an FL, each client has a different amount of training data. Accordingly, each client needs to use different hyperparameters, e.g., batch size and learning rate. As shown in [26], under the non-IID dataset situation, if the batch size for local training is not properly adjusted, performance degradation is inevitable. Hence, the efficiency of the FL can be increased by dynamically controlling the batch size and the learning rate for each client by considering the amount of data $\sum_{𝓁}^{L}s_{k,r}^{𝓁}$ of the clients. By assuming a common number of SGD updates for the clients, an efficient batch size can be obtained. Specifically, in rth round of the proposed scheme, client k uses the value $\left\lfloor \frac{\sum_{𝓁}^{L}s_{k,r}^{𝓁}}{\beta }\right\rfloor$ as its batch size $b_{k,\;r}$, where β is the required number of SGD updates and ⌊⋅⌋ is the floor operator.

Note that the batch size is proportional to the amount of local dataset for each client, which leads to the improvement of the accuracy of the global model. As the clients learn using different batch sizes, it is necessary to control the learning rate for each client for the purpose of converging the global model. Note that when the batch size is small and the learning rate is too large, the loss function of the local model may diverge. Conversely, if the batch size is large and the learning rate is small, the convergence of local learning is too slow. It coincides with the relationship between the learning rate and the batch size shown in [29]. Therefore, the learning rate is adjusted to be proportional to the batch size. In addition, when the learning rate is too large, the model may not converge, accordingly, the learning rate is regulated with arctan function so that the learning rate does not exceed the maximum learning rate $\eta_{\max}$. This process is explained in Algorithm 3.
**Algorithm 3.** DynamicBL. dynamically allocate batch size and learning rate• Input $s_{k,r}$, $\eta_{\max}\;\mathrm{maximum}\;\mathrm{learning}\;\mathrm{rate},\;\beta \;\mathrm{number}\;\mathrm{of}\;\mathrm{SGD}\;\mathrm{update}$ • Output $\mathrm{Batch}\;\mathrm{size}\;b_{k,r},\;\mathrm{learning}\;\mathrm{rate}\;\eta_{k,r}$
1: $b_{k,r}\leftarrow \lfloor \frac{\sum_{𝓁}^{L}s_{k,r}^{𝓁}}{\beta }\rfloor$ 2: $\eta_{k,r}\leftarrow \;\eta_{\max}\times \arctan\left(b_{k,r}\right)$ 3: **return** $b_{k,r},\;\;\eta_{k,r}$


### 4.4. Workflow

**Figure 2:**
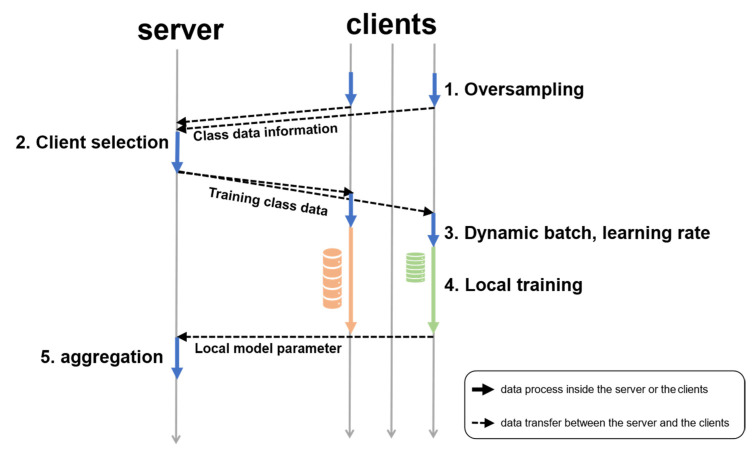
The workflow of the FL with the proposed algorithm, where the orange and green cylinders are the local datasets with different batch sizes. The solid arrow lines mean the data process inside the server or the clients, and the dotted lines mean the data transfer between the server and the clients.

The training procedure of the proposed algorithm consists of local data sampling, client selection and training data allocation, and the control of dynamic batch size and learning rate. This procedure is followed by local training and local model aggregation. This overall process is shown in Figure 2.

- Local data sampling: a client who wants to participate in learning checks the class distribution of the local dataset and proceeds with oversampling, and then sends the data distribution information to the server.
- Client selection and allocation of training data: the server selects the clients to make the class distribution of learning data balanced for each round and delivers the information about the amount of training data to the selected clients.
- Dynamic batch and learning rate control: each client calculates the batch size and learning rate of local learning based on the amount of data it learns.
- Local training: Each client learns a local model using the amount of training data received from the server and the previously calculated batch size and learning rate. After learning, the client sends the local model parameters to the server.
- Aggregation: When the server receives all the selected clients’ local model parameters, it updates the global model parameters using Equation (2). Then repeat until the final round.

## 5. Experiment Results

### 5.1. Experiment Setup

In the performance evaluation of the proposed algorithm, the representative dataset of CIFAR-10 and MNIST are adopted. The deep learning model for this classification task is the convolution neural network (CNN) with two 5 × 5 convolution layers for CIFAR-10, each with 64 and 128 filters. The two convolution layers are followed by a max pool layer, three fully connected layers, and a softmax layer. Then the classification probabilities are derived. We also perform experiments with a simple logistic regression classifier, which we train on the MNIST dataset.

In this evaluation, two baseline algorithms of FedAVG and FedNova [25] are used. FedAVG is a representative algorithm for FL, and FedNova reduces the negative effect of non-IID by normalizing the aggregation step of FedAVG with the number of local updates.

For the CIFAR-10 dataset, the range of the Dirichlet parameter which determines the data distribution of the clients is α∈[0, 0.2], and three test cases are considered according to the setting of Dirichlet distribution. In the case of the MNIST dataset, α=0 and all the clients have only a single class. The maximum number of clients participating in each round, h, is set to 10, and FedAVG and FedNova randomly select 10 clients in each round and conduct global aggregation. The initial oversampling decay exponent δ is set to 0.01, the oversampling decay exponent increment Δ is set to 0.1, and the KLD threshold $\theta_{KLD}$ is set to 0.1 which checks the similarity between the data distribution $v_{r}$ and Uniform distribution.

In the setting for the local model training on CIFAR-10, FedAVG and FedNova set the local epoch, batch size, and learning rate to 5, 64, and 0.1, respectively. The local epoch is set as the same value in FedAVG [3]. For MNIST, local epoch, batch size, and learning rate are 5, 10, and 0.03, respectively. In the proposed algorithm, the number of SGD updates β is set to 3, 25, and 25 for Test Cases 1–3, respectively. The maximum learning rate $\eta_{\max}$ is set to 0.1. It is assumed that the computation capabilities of the clients are equal.

### 5.2. Results on Different Non-IID Data Distribution

As mentioned above, the three different non-IID scenarios of CIFAR-10 Cases 1–3 are considered. In Case 1, α=0 for all the 200 clients, where these clients have only a single class of data. In Case 2, α=0 for 180 out of 200 clients who have only a single class data, and α=0.2 for the remaining 20 clients. On average, a client with α = 0.2 has 6 classes of data, where approximately 4 out of the 6 classes have 26–28% less amount of data than the average amount of data for each class. In Case 3, α = 0.2 for all 100 clients, and it is the most balanced class distribution compared to other test cases. In Case 4, α=0 for 200 clients who have only a single class MNIST data. Table 2 shows the data distribution parameters and experimental parameters for all the test cases. Hyperparameter values are derived experimentally to obtain optimal results.

**Figure 3:**
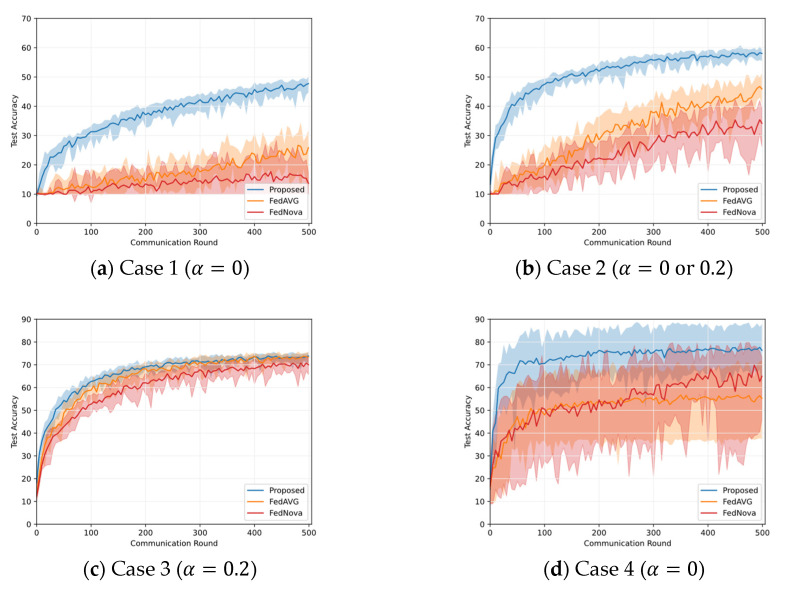
Accuracy comparison between the proposed algorithm and two baseline algorithms (FedAVG, FedNova) on non-IID dataset, where the bold lines represent the average of the accuracy for 10 executions and the light area is the range for the maximum and minimum values. Cases 1–3 are the results of the CIFAR-10 dataset, and Case 4 is the result of the MNIST dataset.

In Figure 3, the average accuracy of the proposed global model is depicted for all the test cases. In obtaining the average accuracy, each algorithm is executed 10 times and averaged. As shown in (a)–(c) of this figure, the achieved accuracy of the proposed algorithm is highest when tested on Case 3 but lowest when tested on Case 1. Note that Case 1 has both severe intra- and inter-client class imbalances. The proposed algorithm achieves 10.4% higher accuracy in Case 2 than in Case 1. As a result, the accuracy of FL decreases when intra-client class imbalance and inter-client class imbalance are very high; however, the accuracy can be improved even when the number of clients having a balanced intra-client class distribution is low. Since Case 3 has the lowest class imbalance, the proposed algorithm achieves the highest accuracy. Figure 3d shows the results of the MNIST dataset, where the proposed algorithm achieves higher accuracy than Case 1, but it has more fluctuation.

Comparing the proposed algorithm with other baseline algorithms, as shown in Figure 3a, in Case 1, the accuracy of the proposed algorithm is improved by 21.8% and 34% compared to FedAVG and FedNova, respectively. Moreover, in this case, the baseline algorithms have a large fluctuation in accuracy from round to round, leading to poor training stability. In addition, when comparing the convergence of the three algorithms, the proposed algorithm converges at a faster rate than the baseline algorithms. In Case 1, since all the clients have only a single class of data, the intra-client class imbalance alleviation method in the proposed algorithm is skipped because this method generates duplicate data elements in non-empty classes. It is noteworthy that the proposed algorithm successfully improves the accuracy of the global model and reduces the variability of the global model without the intra-client class imbalance alleviation method. The baseline algorithms randomly select clients, hence, the sum of the class distributions of the selected clients is imbalanced. As a result, in the baseline algorithms, the accuracy decrement and the high fluctuation are inevitable. On the contrary, the proposed algorithm can improve the accuracy and stability of the global model by applying the inter-client class imbalance method and the dynamic batch size and learning rate control method. In Figure 3b, for Case 2 (α = 0 or 0.2), the proposed algorithm achieves 12.2% and 23.8% accuracy improvements over FedAVG and FedNova, respectively. Moreover, this higher accuracy is achieved within fewer communication rounds and with less fluctuation than FedAVG and FedNova. In the early rounds in Figure 3b, a surge in the accuracy of the proposed algorithm is observed. This rapid accuracy increment in the early rounds is induced by the data oversampling method to mitigate intra-client class imbalance, which enables the learning with more data in the early stage of the learning. As shown in Figure 3c, for Case 3 (α = 0.2), the proposed algorithm does not improve accuracy significantly compared to FedAVG. Unlike Case 1 and Case 2, Case 3 has a balanced class distribution, accordingly, the performance of the proposed algorithm is similar to FedAVG. However, it still achieves about a 4% accuracy improvement over FedNova. In Figure 3d for Case 4, the proposed algorithm shows 21.1% and 11.4% improved accuracy over FedAVG and FedNova, respectively. FedNova performs better than FedAVG on MNIST, and vice versa on CIFAR-10.

### 5.3. Results on Class Imbalance Mitigation

Experiments are conducted to validate the effectiveness of the three core methods which constitute the proposed algorithm. For Cases 1–3 of the CIFAR-10 dataset, which showed the highest performance improvement, we compare it with the baseline algorithms. In the following experiments, the local data oversampling method to alleviate intra-client class imbalance is denoted as ‘data sampling’, the client selection and training data allocation method to alleviate inter-client class imbalance is denoted as ‘client selection’, and the dynamic batch size and learning rate control technique is expressed as ‘dynamic batch’.

**Figure 4:**
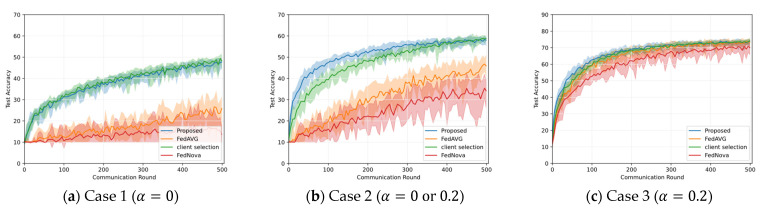
Accuracy comparison among the proposed algorithm, ‘client selection’, and two baseline algorithms on different non-IID situations.

In Figure 4, the accuracy of ‘client selection’ method is compared with the proposed algorithm and two baseline algorithms on Case 1–3. As shown in Figure 4a, for Case 1, the ‘client selection’ method achieves a similar accuracy with the proposed algorithm, and the accuracy improvements of ‘client selection’ over FedAVG and FedNova are 22.5% and 34.6%, respectively. In Figure 4b of Case 2, ‘client selection’ lowers the fluctuation and achieves higher accuracy than FedAVG and FedNova by about 13% and 24.7%, respectively, but it is less accurate than the proposed algorithm. In Case 3, ‘client selection’ achieves a similar accuracy (about 73%) with FedAVG and a 4% higher accuracy than FedNova.

**Figure 5:**
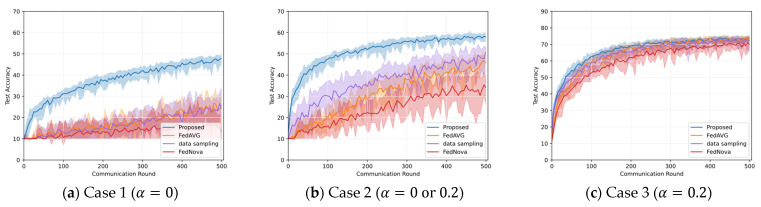
Accuracy comparison among the proposed algorithm, ‘data sampling’, and two baseline algorithms on different non-IID situations.

In Figure 5, the accuracy of ‘data sampling’ is compared with the proposed algorithm and two baseline algorithms in Cases 1–3. In Case 1, ‘data sampling’ is not applied because all the clients have only a single class of data. However, it shows an accuracy improvement of 10.7% compared to FedNova. In Case 2, ‘data sampling’ achieves 4% and 16.4% improvement compared to FedAVG and FedNova, respectively. At the beginning of training, the training accuracy can be improved by increasing the amount of training data through ‘data sampling’. In the latter part of training, the amount of oversampled data is reduced to avoid overfitting, hence, the improvement in accuracy gradually decreases compared to the early part of training. In Case 3, when only ‘data sampling’ is applied, the accuracy improvement is not noticeable because the effect of ‘data sampling’ is evident in scenarios having strong non-IID. Nevertheless, it shows an accuracy improvement of about 2% compared to FedNova.

**Figure 6:**
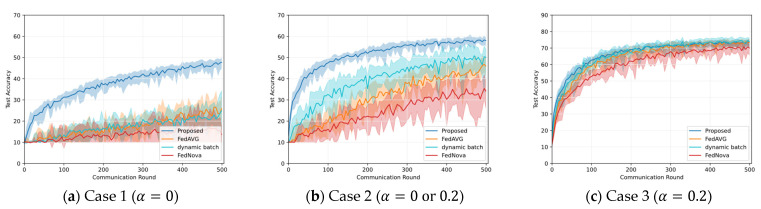
Accuracy comparison among the proposed algorithm, ‘dynamic batch’, and two baseline algorithms on different non-IID situations.

In Figure 6, the accuracy of the ‘dynamic batch’ is compared with the proposed algorithm and two baseline algorithms on Case 1–3. In Cases 1–3, the model fluctuations are similar to the baseline algorithms. In Case 1 and 3, the accuracy is similar to that of FedAVG; however, compared to FedNova, the proposed algorithm shows 11.2% and 4% improvement in Case 1 and Case 3, respectively. In Case 2, the accuracy is improved by about 4% and 16% compared to FedAVG and FedNova, respectively. Compared to Case 1, in Case 2, the clients have various class distributions, hence, if the batch size and the learning rate for each client are not properly adjusted, it is difficult to extract high performance, and it makes the effect of ‘dynamic batch’ conspicuous. In Case 3, the clients have more classes than in Case 2, accordingly, the contribution of the ‘dynamic batch’ in improving accuracy is relatively small.

### 5.4. Amount of Training Data

**Figure 7:**
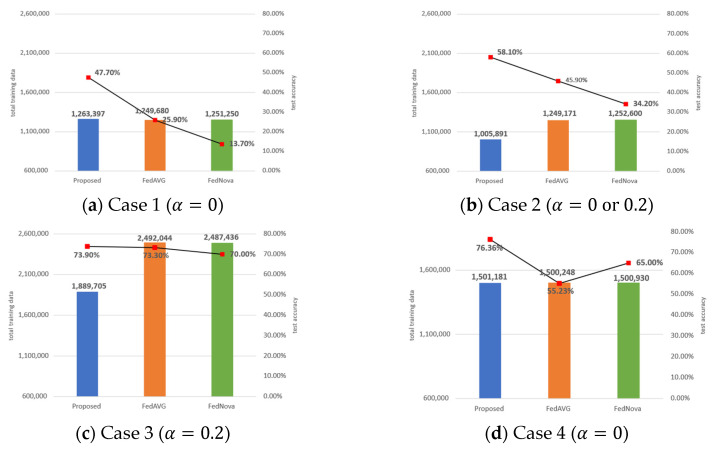
Amount of training data (bar graph) and accuracy (red dot) comparison among the proposed algorithm, FedAVG, and FedNova on different non-IID situations. Cases 1–3 are the results of the CIFAR-10 dataset, and Case 4 is the result of the MNIST dataset.

Since the proposed algorithm determines the amount of local training data for the clients on each round, the clients can learn using only a subset of their local dataset. In Case 1–4, the total amount of data used for the proposed algorithm is compared to FedAVG and FedNova, and the results are shown in Figure 7.

As shown in Figure 7a, the amount of data samples used for the proposed algorithm is about 1,263,000, and it is roughly 1% more than the amount of data for FedAVG. In Case 1, since all the clients have only a single class, ‘data sampling’ is not applied, hence, the amount of training data does not increase. However, compared to FedAVG which randomly selects clients, the proposed algorithm is prone to select clients with more data to train. Thus, as shown in Figure 7a, the proposed uses 1% more data. In addition, in Case 1, most of the local datasets are used in training. For this reason, in Case 1, the number of training data is similar to FedAVG and FedNova, which uses all of the client’s local data in training. However, it should be noted that even though the proposed algorithm uses a small amount of more data (about 1% more) than the baseline algorithms, the accuracy improvement is remarkably high by 21.8% and 34.4% compared to FedAVG and FedNova, respectively.

In Figure 7b for Case 2, the proposed algorithm uses about 1,005,000 data samples, and the efficiency of the proposed algorithm is clearly shown in this figure. Specifically, the proposed algorithm uses 19% less data than the baseline algorithms, while the achieved accuracy is higher by 12.2% and 23.8% compared to FedAVG and FedNova, respectively. In Case 2, ‘data sampling’ is applied; however, the amount of oversampled data is quickly reduced to avoid overfitting. This mechanism also minimizes the potential burden of increasing the amount of data to train.

In Figure 7c for Case 3, the accuracy difference between the proposed algorithm and FedAVG is negligible at 0.6%, and between the proposed algorithm and FedNova, it is not high at 3.9%. However, the proposed algorithm uses 24% less amount of data than FedAVG and FedNova, and it is a huge gap.

In Figure 7d for Case 4, all three algorithms learn using a similar number of training data about 1,500,000. However, when comparing the test accuracy of the proposed algorithm with FedAVG and FedNova, it shows 21.1% and 11.4% improved results, respectively.

Figure 7 shows the adaptability of the proposed in improving accuracy and reducing the amount of training data. More specifically, in a severe non-IID situation like Case 1, the proposed algorithm mainly focuses on increasing the accuracy rather than reducing the used training data volume as shown in Figure 7a. When the level of non-IID is low like in Case 3, the proposed algorithm focuses on reducing the training data volume rather than increasing the accuracy as shown in Figure 7c. When the level of non-IID is medium like in Case 2, both the accuracy and the amount of training data are improved as shown in Figure 7b. Through the amount of training data used in Case 1–3, it is confirmed that, on average, the proposed algorithm achieves higher accuracy by using lower computing resources than FedAVG and FedNova.

### 5.5. Average Number of Clients

**Figure 8:**
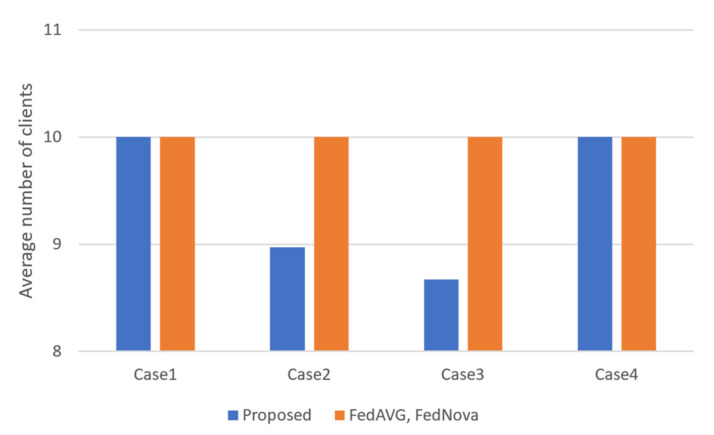
Average number of clients participating in the learning on different non-IID situations.

The average number of clients participating in the learning on each round is depicted in Figure 8. FedAVG and FedNova randomly select a fixed number of clients on every round, while the proposed algorithm can terminate the client selection process before the number of the selected client reaches the maximum h if the data information $v_{r}$ for training becomes close enough to Uniform distribution. In Case 1 and Case 4, since all the clients have only a single class, the maximum number of clients must be selected to make $v_{r}$ similar to Uniform distribution. In Cases 2 and 3, higher test accuracy is achieved even though fewer clients participate in the learning than FedAVG and FedNova. In FL, the reduced number of clients results in the reduced usage of communication resources. Therefore, it is confirmed that the proposed algorithm uses lower communication resources than the baseline algorithms.

## 6. Conclusions

In FL, if the clients’ local data distribution is non-IID, the accuracy and learning efficiency of the global model decreases. To solve this problem, the intra-client class imbalance is alleviated through local data sampling, and inter-client class imbalance is alleviated by selecting the clients and determining the amount of data to be used for training, which makes the aggregate of the training data class distributions balanced on every round. In addition, more efficient local learning is possible by dynamically determining the batch size and learning rate reflecting the amount of training data. The proposed algorithm achieves faster convergence speed and higher accuracy with lower computing and communication resource usage than existing algorithms in non-IID environments.

## Figures and Tables

**Table 1:** Notation and definitions.

Notation	Definition
K	Client index set
*r*	Round index
$\theta_{\mathrm{KLD}}$	Kullback–Leibler divergence threshold
h	Maximum number of selected clients
L	Number of classes
δ	Oversampling exponent
$b_{k,r}$	Batch size of client k at round r
$\eta_{k,r}$	Learning rate of client k at round r
$w_{r}$	Global model parameter at round r
$w_{k,r}$	Local model parameter of client k at round r
$D_{k}$	Local dataset of client k
K	Number of clients
${ℬ}_{k}$	Mini batch set for client *k*
$f_{k}\left(\cdot \;;\;\cdot \right)$	Local loss function of client k
α	Dirichlet distribution control parameter
$n_{k}$	Class data volume for client k
*t_k_*	Average amount of class data for client k
$s_{k,r}$	Class training data volume for client k at round r
***v**_r_*	Class training data volume at round *r*
β	Number of SGD updates
*η*_max_	Maximum learning rate

**Table 2:** Distribution setup and experiment parameters.

	Distribution Setup			Experiment Parameter					
Datasets	Case	K	α	Sampling	Client Selection	Dynamic Batch		Local Training	
				$\theta_{\mathrm{over}}$	$\theta_{KLD}$	$\eta_{\max}$	𝛽	h	epoch
CIFAR-10	1	200	0	0.1	0.1	0.1	3	10	5
	2	200	0 or 0.2	0.1	0.1	0.1	25	10	5
	3	100	0.2	0.1	0.1	0.1	25	10	5
MNIST	4	200	0	0.1	0.1	0.1	25	10	5

## Data Availability

Publicly available datasets were analyzed in this study. This data can be found here: [https://www.cs.toronto.edu/~kriz/cifar.html, https://yann.lecun.com/exdb/mnist/].

## References

[1] Cisco (2020). Cisco Annual Internet Report (2018–2023).

[2] McMahan B., Ramage D. (2018). Research Scientists. Federated Learning: Collaborative Machine Learning without Centralized Training Data. https://ai.googleblog.com/2017/04/federated-learning-collaborative.html.

[3] McMahan B., Moore E., Ramage D., Hampson S., Arcas B.A. Communication-efficient learning of deep networks from decentralized data. Proceedings of the Artificial Intelligence and Statistics (AISTATS 2017).

[4] Nishio T., Yonetani R. Client selection for federated learning with heterogeneous resources in mobile edge. Proceedings of the 2019 IEEE International Conference on Communications (ICC 2019).

[5] Wang S., Tuor T., Salonidis T., Leung K.K., Makaya C., He T., Chan K. (2019). Adaptive Federated Learning in Resource Constrained Edge Computing Systems. IEEE J. Sel. Areas Commun..

[6] Wang Z., Xu H., Liu J., Huang H., Qiao C., Zhao Y. Resource-Efficient Federated Learning with Hierarchical Aggregation in Edge Computing. Proceedings of the IEEE INFOCOM 2021—IEEE Conference on Computer Communications.

[7] Sattler F., Wiedemann S., Muller K.-R., Samek W. (2020). Robust and Communication-Efficient Federated Learning from Non-i.i.d. Data. IEEE Trans. Neural Netw. Learn. Syst..

[8] Zhao Y., Gong X. Quality-Aware Distributed Computation and User Selection for Cost-Effective Federated Learning. Proceedings of the IEEE INFOCOM 2021—IEEE Conference on Computer Communications Workshops (INFOCOM WKSHPS).

[9] Ma Z., Xu Y., Xu H., Meng Z., Huang L., Xue Y. (2021). Adaptive Batch Size for Federated Learning in Resource-Constrained Edge Computing. IEEE Trans. Mob. Comput..

[10] Shi D., Li L., Wu M., Shu M., Yu R., Pan M., Han Z. (2022). To Talk or to Work: Dynamic Batch Sizes Assisted Time Efficient Federated Learning over Future Mobile Edge Devices. IEEE Trans. Wirel. Commun..

[11] Li Q., Diao Y., Chen Q., He B. Federated Learning on Non-Iid Data Silos: An Experimental Study. Proceedings of the 2022 IEEE 38th International Conference on Data Engineering (ICDE).

[12] Zhao Y., Li M., Lai L., Suda N., Civin D., Chandra V. (2018). Federated Learning with Non-IID Data. arXiv.

[13] Krizhevsky A., Hinton G. (2009). Learning Multiple Layers of Features from Tiny Images.

[14] Li D. (2012). The MNIST Database of Handwritten Digit Images for Machine Learning Research [Best of the Web]. IEEE Signal Process. Mag..

[15] Li T., Sahu A.K., Zaheer M., Sanjabi M., Talwalkar A., Smith V. (2018). Federated Optimization in Heterogeneous Networks. arXiv.

[16] Shoham N., Avidor T., Keren A., Israel N., Benditkis D., Mor-Yosef L., Zeitak I. (2019). Overcoming forgetting in federated learning on non-iid data. arXiv.

[17] Rizk E., Vlaski S., Sayed A.H. Optimal Importance Sampling for Federated Learning. Proceedings of the ICASSP 2021—2021 IEEE International Conference on Acoustics, Speech and Signal Processing (ICASSP).

[18] Kopparapu K., Lin E. (2020). Fedfmc: Sequential efficient federated learning on non-iid data. arXiv.

[19] Briggs C., Fan Z., Andras P. Federated Learning with Hierarchical Clustering of Local Updates to Improve Training on Non-IID Data. Proceedings of the 2020 International Joint Conference on Neural Networks (IJCNN).

[20] Duan M., Liu D., Chen X., Tan Y., Ren J., Qiao L., Liang L. Astraea: Self-Balancing Federated Learning for Improving Classification Accuracy of Mobile Deep Learning Applications. Proceedings of the 2019 IEEE 37th International Conference on Computer Design (ICCD).

[21] Auer P., Cesa-Bianchi N., Fischer P. (2002). Finite-time analysis of the multiarmed bandit problem. Mach. Learn..

[22] Zhang W., Wang X., Zhou P., Wu W., Zhang X. (2021). Client Selection for Federated Learning With Non-IID Data in Mobile Edge Computing. IEEE Access.

[23] Yang M., Wang X., Zhu H., Wang H., Qian H. Federated Learning with Class Imbalance Reduction. Proceedings of the 2021 29th European Signal Processing Conference (EUSIPCO).

[24] Malandrino F., Chiasserini C.F. (2021). Federated Learning at the Network Edge: When Not All Nodes Are Created Equal. IEEE Commun. Mag..

[25] Wang J., Liu Q., Liang H., Joshi G., Poor H.V. (2020). Tackling the Objective Inconsistency Problem in Heterogeneous Federated Optimization. Adv. Neural Inf. Process. Syst. (NeurIPS).

[26] Zhang J., Guo S., Qu Z., Zeng D., Zhan Y., Liu Q., Akerkar R. (2022). Adaptive Federated Learning on Non-IID Data with Resource Constraint. IEEE Trans. Comput..

[27] Hsu T.M.H., Qi H., Brown M. (2019). Measuring the effects of non-identical data distribution for federated visual classification. arXiv.

[28] Kullback S., Leibler R.A. (1951). On Information and Sufficiency. Ann. Math. Stat..

[29] Goyal P., Dollár P., Girshick R.B., Noordhuis P., Wesolowski L., Kyrola A., Tulloch A., Jia Y., He K. (2017). Accurate, Large Minibatch SGD: Training ImageNet in 1 h, CoRRabs/1706.02677. https://dblp.org/db/journals/corr/corr1706.html#GoyalDGNWKTJH17.

